# *In vivo* inducing collagen regeneration of biodegradable polymer microspheres

**DOI:** 10.1093/rb/rbab042

**Published:** 2021-07-15

**Authors:** Yixin Zhang, Hanwen Liang, Qian Luo, Jianlin Chen, Nan Zhao, Wenxia Gao, Yuji Pu, Bin He, Jing Xie

**Affiliations:** 1 School of Smart Health, Chongqing College of Electronic Engineering, Chongqing 401331, China; 2 School of Laboratory Medicine, Sichuan Provincial Engineering Laboratory for Prevention and Control Technology of Veterinary Drug Residue in Animal-origin Food, Chengdu Medical College, Chengdu 610500, China; 3 Puliyan (Nanjing) Medical Science & Technology Co. LTD, Nanjing 211500, China; 4 College of Chemistry and Materials Engineering, Wenzhou University, Wenzhou 325027, China; 5 National Engineering Research Center for Biomaterials, Sichuan University, Chengdu 610064, China; 6 Department of Stomatology, The First Affiliated Hospital of Wenzhou Medical University, Wenzhou 325000, China

**Keywords:** biodegradable polymers, microspheres, collagen regeneration, dermal fillers

## Abstract

Biodegradable polymer particles have been used as dermal fillers for pre-clinical and clinical trials. The impact of material properties of polymers is very important to develop products for aesthetic medicine such as dermal fillers. Herein, eight biodegradable polymers with different molecular weights, chemical compositions or hydrophilic-hydrophobic properties were prepared and characterized for systematical study for aesthetic medicine applications. Polymer microspheres with 20–100 μm were prepared. The *in vitro* degradation study showed that poly (L-lactic-*co*-glycolic acid) 75/25 microspheres degraded the fastest, whereas poly (L-lactic acid) (PLLA) microspheres with intrinsic viscosity of 6.89 ([*η*] = 6.89) with the highest molecular weight showed the slowest degradation rate. After these microspheres were fabricated dermal fillers according to the formula of Sculptra^®^, they were injected subcutaneously into the back skin of rabbits. *In vivo* results demonstrated that the degradation rate of microspheres strongly correlated with the foreign body reaction and collagen regeneration was induced by microspheres. The microspheres with faster degradation rate induced inflammatory response and the collagen regeneration maintained in shorter time. PLLA ([*η*] = 3.80) microsphere with a moderate molecular weight and degradation rate could strongly regenerate Type I and III collagen to maintain a long-term aesthetic medicine effect. These properties of size, morphology and degradation behavior would influence the foreign body reaction and collagen regeneration.

## Introduction

Skin aging, an inevitable physiological process, causes fundamental changes in the skin appearance and structure. Dermal fillers specialize in repairing soft tissue volume loss and deep static wrinkles or folds, having advantages of simple operation, small trauma, short recovery time and obvious repair effect, etc. [1, 2] When compared with traditional dermal fillers that only have functions of replacement or filling, the new-generation dermal fillers like Sculptra^®^, whose main ingredient is poly (L-lactic acid) (PLLA) microspheres, has biological stimulation effect to stimulate the regeneration of endogenous collagen and dermal fibrocytes after injection [3, 4]. The effect of PLLA microspheres on deep wrinkles or folds such as crow’s feet, nasolabial furrows and puppet strings is significant and could maintain for 18–25 months [5], and on forearm area could even over 28 months [6]. A typical bottle of Sculptra^®^ contains 150 mg of PLLA microsphere freeze-dried powder, 90 mg of sodium carboxymethyl cellulose (CMC) and 127.5 mg of mannitol [7]. CMC is used as an excitant to increase the viscosity of the system and avoid the rapid deposition of microspheres, which will affect the injection or cause uneven distribution in the body [8, 9]. Mannitol is a lyophilized protective agent and has antioxidant activity, scavenging free radicals in the body, inhibiting the rapid degradation of the filler material and reducing the risk of edema after injection [10].

Biodegradable polymer microspheres are believed to be active components that stimulate collagen regeneration. Upon injection, the microsphere is first coated with lymphocytes such as macrophages and giant cells, causing a mild inflammatory response for several months. Then, the microsphere is gradually degraded into carbon dioxide and water and new collagen and other connective tissue collagens are formed [11]. Lemperle *et al*. [12] evaluated the foreign body reaction caused by subcutaneous injection of New-fill (dermal filler of PLLA microspheres, Sculptra^®^) into the palmar skin of human forearm. The results showed that PLLA microspheres could be observed in soft tissue 2 weeks after injection. After 3 months, PLLA microsphere remained spherical and was surrounded by macrophages and some lymphocytes. After 6 months, porous structure or irregular shape appeared on the surface of the microsphere, with macrophages and giant cells around. PLLA microspheres ‘disappeared’ at 9 months. In addition, other researchers focused on the collagen types stimulated by PLLA microspheres. Type I collagen, mainly exists in adult skin, tendon and bone, is a relatively hard collagen. Type III collagen, mainly in the baby’s skin or vascular intima and bowel, is an elastic collagen. Type I collagen increases with age, whereas Type III collagen shrinking. Eventually, the synthesis rate of Type III collagen could not catch up with its loss rate, leading to wrinkles and relaxation, thus stimulating regeneration of Type III collagen can effectively relieve skin aging. Goldberg *et al.* [13] evaluated tissue reaction to Scupltra^®^ and found that after injected for 3 and 6 months, Type I and Type III collagens were observed and increased significantly; normal collagen increase was shown after 12 months.

As a biodegradable polymer, PLLA has been widely used in various biomedical applications for decades [14–18]. At present, a large number of literatures have been focused on the evaluation of the foreign body reaction caused by Sculptra^®^ in animals and human bodies and the comparison of *in vivo* effects between Sculptra^®^ and other dermal fillers [13, 19–21]. However, few studies have been carried out to explore the effect of polymer materials (such as molecular weights, compositions, different polymer structures and architectures) on the biodegradation, inflammatory response, and collagen regeneration profiles. Based on the current situation of PLLA-based medical and aesthetic products, in this study, we developed biodegradable polymers with different molecular weights (PLLA), chemical structures (poly (L-lactic-*co*-glycolic acid) [PLLGA]) and polymer architectures to evaluate their biocompatibility, biodegradability and potentials as dermal fillers. Polymer microspheres with size of 5–100 μm were prepared and injected into the back of rabbits. A 13-month observation of *in vivo* degradation and inflammatory response, collagen regeneration was performed.

## Experimental

### Materials and animals

L-lactide (L-LA, 99%, Purac) and glycolide (GA, 99%, Purac) were recrystallized in ethyl acetate. PEG (*M*_n_ = 2 kDa, Sigma-Aldrich), PLLA ([*η*] = 3.80, Purac), tin(II)2-ethylhexanoate (Sn(Oct)_2_, 99.5%, Sigma-Aldrich), poly (vinyl acid) (PVA, Dongren Chemical Co.), CMC (90 kDa, Aladdin), mannitol (Adamas) and ethanol (Kelong Chemical Co.) were used as received. DL-lactic acid, chloroform (CHCl_3_) and dimethyl sulfoxide (DMSO) were purchased from Chengdu Kelong Chemical Co. (Chengdu, China) and dried over CaH_2_ and distilled under vacuum. Dulbecco’s modified Eagle’s medium (DMEM), fetal bovine serum (FBS), penicillin-streptomycin, and 3-(4,5-dimethylthiazol-2-yl)-2,5-diphenyl tetrazolium bromide (MTT) were used for cell study. Female New Zealand white rabbits were supplied by Byrness Weil biotech Ltd. (Chongqing, China). All animal experiments were performed following the procedure approved by Institutional Animal Care and Use Committee, Chengdu Medical College.

### Characterizations

^1^H nuclear magnetic resonance (^1^H NMR) spectra were recorded on a Bruker Avance NMR spectrometer (400 MHz) using CDCl_3_ as solvent with 0.5% tetramethyl silane as the internal standard. Gel permeation chromatography (GPC) measurement was accomplished on an Agilent, 1100 Series, including a Waters 717 model auto sampler, a 2414 refractive index detector and a Waters instrument equipped with a model 1515 pump. Chloroform was utilized as the mobile phase at a flow rate of 1.0 ml/min at 25°C. Ubbelohde viscometer was used to measure the intrinsic viscosity in order to calculate the viscosity-average molecular weight (*M*_v_) of the polymers and chloroform was used as solvent at 25°C. Scanning electron microscopy (SEM, s4800, Hitachi Ltd., Tokyo, Japan) was used to observe the morphology and size of microspheres. Gamma (γ) irradiation plant (FJx424, Beijing Institute of Nuclear Engineering, Beijing, China) was used for irradiation sterilization.

### Synthesis of PLLA with different molecular weight

PLLA was synthesized by ring opening polymerization of L-LA by using DL-lactic acid as initiator and Sn(Oct)_2_ as catalyst. The molecular weights of PLLAs can be adjusted by varying the feeding molar ratios of monomer/initiator. Briefly, 20 g of L-LA and certain mass of DL-lactic acid were dried under vacuum in a polymerization tube for 2 h, and 1 ml of Sn(Oct)_2_ in toluene (0.01 wt.%) was added. The tube was sealed and immerged in an oil bath at 130°C for 48 h. Then, the product was dissolved in CHCl_3_ and the solution was precipitated in excessive ethanol. PLLA was obtained after filtration and drying under vacuum. The detailed data of initiators and monomers used for the synthesis of PLLA with different molecular weights were listed in Supplementary Table S1.

### Synthesis of PLLGA 75/25 and PLLGA 85/15

The synthesis of PLLGA 75/25 and PLLGA 85/15 was similar with the synthetic process of PLLA except that GA was introduced into the system and the molar ratios between L-LA and GA were 75/25 and 85/15, respectively. The detailed amounts of initiators and monomers used for the synthesis of PLLGA were also listed in Supplementary Table S1.

### Synthesis of triblock copolymer PLLA-PEG-PLLA

The synthesis of PLLA-PEG-PLLA was also similar with the synthetic process of PLLA except that PEG was used as a macro-initiator. The detail data during the process were listed in Supplementary Table S1.

### Preparation of polymeric microspheres

Blank polymer microspheres were prepared by a classical emulsion-solvent evaporation method. Briefly, 10 ml of CHCl_3_ with certain mass of polymer was dropped into 100 ml of PVA aqueous solution and emulsified by mechanical stirrer for 3 h or homogenizer for 30 min. After the evaporation of CHCl_3_, the mixture was centrifuged (4000 rpm, 5 min) and washed with water for two times to remove residual PVA. The microsphere suspension in deionized water was lyophilized to obtain polymer microspheres. Since the size of microspheres were affected seriously by some parameters such as viscosity of oil phase, concentration of stabilizer or emulsifier, shear force and temperature, [22–24] some key parameters for the microspheres with appreciated size were shown in Supplementary Table S2.

### *In vitro* degradation behaviors of microspheres

Polymer microspheres (30 mg) irradiated at a dose of 25 kGy were dispersed in 10 ml of phosphate buffer saline (PBS, pH = 7.4) at 37°C and the rotating speed of shaking table was set at 120 rpm. The PBS was replaced every 2 weeks. At each time point, the corresponding solution was centrifuged (4000 rpm, 5 min) for three times to remove residual salts and degradation products. After freeze-drying, the molecular weights of samples were measured by GPC and the morphology of microspheres was observed by SEM.

### MTT assay

MTT assay was carried out to evaluate the biocompatibility of these polymer microsphere dermal fillers prepared according to the Sculptra^®^ formula. L929 cells seeded in 96-well plates were cultured in DMEM with 1% v/v penicillin/streptomycin and 10% v/v FBS at 37 °C, in a 5% CO_2_ atmosphere for 12 h. Then, cells were cultured with medium containing different concentrations of dermal fillers (calculated as polymeric concentration) for 24, 48 and 72 h (the corresponding cell density was 5 × 10^3^, 8 × 10^3^ and 1 × 10^4^ cells per well, respectively). The cells were then treated with MTT for 4 h and DMSO was added to dissolve the formazan crystals. Finally, the UV absorbance at 490 nm [25, 26] was measured by a microplate reader and the cell viability was calculated.

### *In vivo* effect of aesthetic medicine

Microspheres (200 mg) were dispersed into certain volume of aqueous solution containing 122 mg of CMC and 165.8 mg of mannitol according to the Sculptra^®^ formula. After freeze-drying, the solid was irradiated by 25 kGy γ-ray for sterilization. Then, the solid was dispersed into 6.67 ml of sterilized saline to obtain a microsphere concentration of 30 mg/ml. After the mixture was left standing over 24 h to make CMC sufficiently hydrated, it was shaken and vortexed well before use.

After the female rabbits (about 2 kg) had unhaired from their back, each of them was permanently marked five 1.5 cm^2^ grids in different places on the back. The five different kinds of dermal fillers selected were subcutaneously injected into different marked grids (0.2 ml of fillers/grid), respectively, and three rabbits as three duplicated samples were set for each time point (each kind of dermal filler was injected on the backs of three rabbits for each time point). A 0.2 ml of saline was subcutaneously injected into the back of another rabbit. After the injections, the rabbits’ growth condition was regularly observed and the appearance of erythema or edema on the back skin was checked. The tissue samples around the injection area were obtained for histological examination at 0.5, 1.5, 2.5, 4, 6, 9 and 13 months post injection. The samples were fixed immediately for hematoxylin-eosin (H&E), Masson staining as well as CD68, Type I and Type III collagens immunofluorescence staining. The collagen immunofluorescence staining images were processed by ImageJ to perform the semi-quantitative analysis. Additionally, the inflammatory response grade classifications of microspheres at different time points were recorded according to the classification of foreign body reaction established by Duranti *et al.* [20].

Grade 0: No inflammatory reaction;

Grade I: slight reaction with a few inflammatory cells;

Grade II: clear inflammatory reaction with one or two giant cells;

Grade III: fibrous tissue with inflammatory cells, lymphocytes, and giant cells; and

Grade IV: granuloma with encapsulated implants and clear foreign body reaction.

## Results and discussion

### Preparation and characterization of polymer microspheres

^1^H NMR spectra of PLLA, PLLGA75/25, PLLGA85/15 and PLLA-PEG-PLLA are displayed in Supplementary Fig. S1. The molecular weights of these polymers were measured by GPC and the intrinsic viscosity [η] measured through Ubbelohde viscometer to calculate the *M*_v_ in terms of the Mark–Houwink empirical formula (PLLA: [*η*] = 4.7 × 10^−4^*M*_v_^0.67^; PLLGA: [*η*] = 3.3 × 10^−4^*M*_v_^0.69^) offered by Purac Ltd. Co. were listed in Table 1.

**Table 1.** Intrinsic viscosities, molecular weights and size distributions of polymers

	[*η*] (dl/g)	*M*_v_ (kDa)	*M*_n_ (kDa)	PDI^b^	Size (μm)
PLLA-0.62	0.62	45.4	32.2	1.41	20–80
PLLA-1.18	1.18	119	108	1.5	30–90
PLLA-1.39	1.39	152	110	1.53	20–85
PLLA-3.80	3.80	604	208	1.93	20–90
PLLA-6.89	6.89	1.65 × 10^3^	289	2.19	5–100
PLLGA 75/25	1.10	128	115	1.41	20–75
PLLGA 85/15	1.32	166	128	1.53	20–75
PLLA-PEG-PLLA	0.70	≈54.4^a^	68.3	1.38	50–100

^a^*M*_v_ of PLLA-PEG-PLLA was calculated approximately according to the Mark–Houwink empirical formula of PLLA since their chemical structures are similar.^b^ Polymer dispersity index

**Figure 1.**
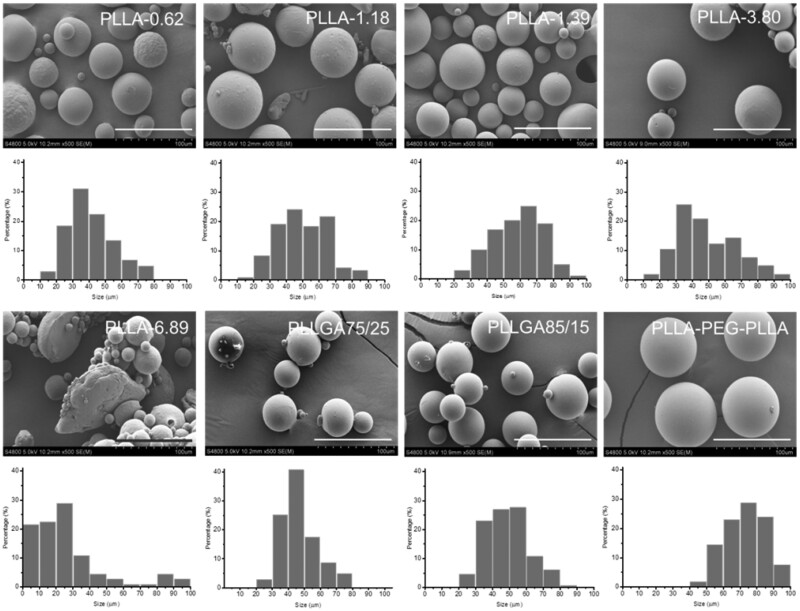
SEM images and SEM size distributions of different polymeric microspheres. The scale bar is 100 µm.

Since the size of polymer microspheres was one of the most important indexes that guaranteed the safety of use, after plenty of experiments, each kind of microspheres with suitable size range was prepared and the data obtained from SEM images and analyzed by Image Pro software (Table 1). The relative parameters during the preparation of these microspheres were presented in Supplementary Table S2. As shown in Table 1, the sizes of all the microspheres except PLLA-6.89 were in the range from 20 to 100 μm, which could not only protect microspheres from recognition and clearance by immune cells but also avoid clogging a needle [27]. The morphology and size distribution of these microspheres were studied by SEM and displayed in Fig. 1. All the microspheres except PLLA-6.89 showed a spherical shape and smooth surface. The sizes of most of these microspheres were in the range of 30–70 μm. PLLA-6.89 microspheres had irregular flaky or table-shaped particles with rough surface and its size distribution was wider than others due to the high viscosity of oil-phase caused by ultra-high-molecular weight of PLLA during the preparation process. What’s more, the size distributions of PLLA-PEG-PLLA, PLLGA 75/25 and PLLGA 85/15 microspheres were narrower than those of other microspheres because of lower molecular weight of PLLA-PEG-PLLA and lower crystallinity of PLLGA, which facilitated that the low viscosity droplets were evenly sheared during the emulsion phase.

### Irradiation effect on the molecular weight and microsphere morphology

The high-energy gamma rays would break the molecular chain of polymer during the process of irradiation for sterilization. Therefore, it is necessary to explore the influence of irradiation dose on molecular weight of polymers and microsphere morphology. The changes of their *M*_v_ and [η] before and after irradiation of 25 and 50 kGy γ-rays are shown in Table 2. The *M*_v_ of these polymers reduced about 20–40% at the dose of 25 kGy while their *M*_v_ reduced about 70–90% at 50 kGy, suggesting that higher dose irradiation could lead to more intense bond cleavage of polymers. The molecular weight and dispersity of polymers before and after irradiation were further studied by GPC. As shown in Supplementary Table S3, the *M*_n_ of PLLA-3.80 decreased by 60% and 89% after irradiation of 25 and 50 kGy, respectively. Higher dose irradiation led to more cleavage of chemical bonds in the backbone of polymers, thereby a lower molecular weight. Meanwhile, the molecular weight dispersity was slightly reduced after irradiation treatment. The reason might be that the irradiation broke the chains of PLLA-3.80 and resulted in closer chain length, thus, the PDI was reduced after irradiation.

**Table 2.** Molecular weights and intrinsic viscosities of polymeric microspheres before and after ray irradiation^a^

	PLLA-0.62	PLLA-1.18	PLLA-1.39	PLLA-3.80	PLLA-6.89	PLLGA 75/25	PLLGA 85/15	PLLA-PEG- PLLA
[*η*] (0 kGy)	0.62	1.18	1.39	3.80	6.89	1.10	1.32	0.70
[*η*] (25 kGy)	0.53	1.02	1.17	2.70	5.38	0.92	1.14	0.60
[*η*] (50 kGy)	0.20	0.54	0.57	0.93	1.15	0.48	0.55	0.21
*M*_v_ (0 kGy)	45.4	119	152	680	1.65 × 10^3^	128	166	54.4
*M*_v_ (25 kGy)	36.3	95.0	118	408	1.14 × 10^3^	98.4	134	43.2
*M*_v_ (50 kGy)	8.39	37.0	40.0	83.2	114	38.3	46.7	9.02

^a^The units of [*η*] and *M*_v_ are dl/g and kDa, respectively.

**Figure 2.**
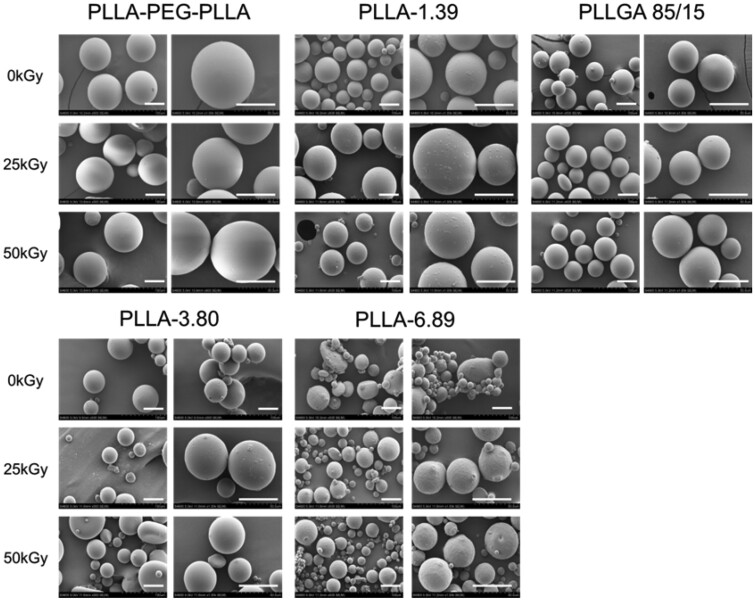
SEM images of polymeric microspheres before and after irradiation. The scale bar is 50 µm.

The SEM images (Fig. 2) illustrated that the morphology of these microspheres was hardly influenced by the ray irradiation even at 50 kGy. The reason was that the degradation of PLLA and PLLGA follows the bulk erosion mechanism [28, 29] and the irradiation dose of 25 and 50 kGy did not reach the critical point to disintegrate them. Therefore, the microsphere morphology remained unchanged but the molecular weight decreased. In addition, the high molecular weight of polymers also ensured that the molecular weight would not fall too low to change the morphology.

### *In vitro* degradation of polymeric microspheres after irradiation

**Figure 3.**
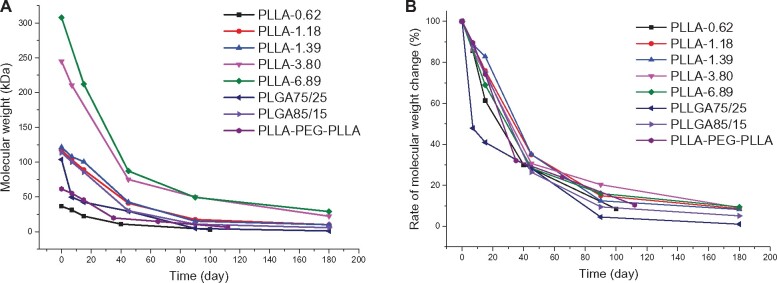
The molecular weights (**A**) and molecular weight percentage (**B**) of polymers over time in the PBS buffer solution (pH 7.4) at 37°C.

After *in vivo* injection, the polymer microspheres would be gradually degraded and absorbed owing to the hydrolysis and/or enzymolysis of ester bonds. To simulate the *in vivo* degradation behavior of these polymer microspheres, microsphere degradation in PBS buffer solution (pH 7.4) at 37°C was investigated. The molecular weight changes of polymers over time were monitored by GPC. Figure 3A displays the molecular weight (*M*_p_) changes of different polymers. The degradation rate was faster in Days 0–90 than in Days 90–180. Of note, PLLGA75/25 having a similar molecular weight to those of PLLA-1.18, PLLA-1.39 and PLLGA85/15 showed a significantly faster degradation due to the amorphous state of the chain. Figure 3B depicts the molecular weight percentage changes to omit the influence of the molecular weight on the degradation. All polymers showed similar molecular weight percentage loss except that PLLGA75/25 showed more molecular weight loss percentage in the first 40 days; about 80% molecular weight loss was observed at 90th day for all polymers, suggesting the rapid degradation potential *in vivo*. The degradation rate of PLLGA75/25 was higher than that of PLLAs because the introduction of GA segment in the chain affected the crystallization of the original PLLA segment, which facilitates the water contact and the subsequent hydrolysis degradation [30]. The degradation rate of PLLA-PEG-PLLA microspheres was slightly faster than that of PLLAs, and its molecular weight reduced to 66.7% of pristine molecular weight after 35 days. This was because the hydrophilic PEG segment embedded in the main chain allowed water to penetrate into the polymer matrix of the microspheres more easily, thus facilitating the hydrolysis process. Above all, the degradation rate order was PLLA-0.62 > PLLA-PEG-PLLA > PLLGA75/25 > PLLA-1.18 ≈ PLLGA85/15 ≈ PLLA-1.39 > PLLA- 3.80 > PLLA-6.89. Besides of the susceptibility to water, polyesters can be degraded by esterase *in vivo*. Some researchers also studied the *in vitro* degradation behaviors of PLLA and PLLGA by esterase. The results illustrated that the weight loss rate in esterase group was about seven times faster than that in PBS group [31].

**Figure 4.**
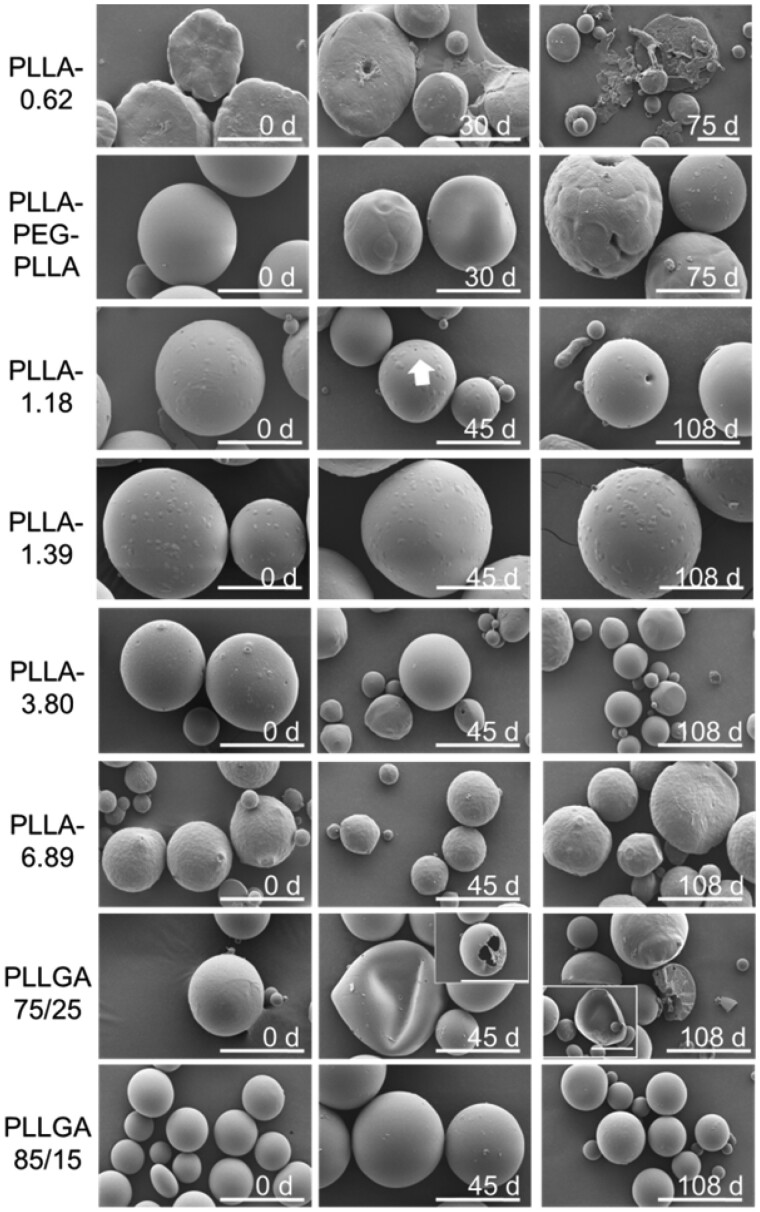
Morphology changes of the polymeric microspheres in the PBS buffer solution (pH 7.4) at different time points. The white arrow pointed a single deep hole on the surface of microspheres. The scale bar is 50 µm.

In order to further study the *in vitro* degradation behavior of these polymeric microspheres, the size and morphological changes of these microspheres were observed by SEM (Fig. 4). PLLA-1.39, PLLA-3.80 and PLLA-6.89 microspheres did not show significant morphology change even after 108 days due to the high molecular weights and the bulk erosion degradation mechanism [32]. Nevertheless, the morphology of other polymer microspheres changed with the extension of time. For example, on the 45th day, a few small holes appeared on the surface of the PLLGA75/25 microspheres, and about 50% of the microspheres collapsed. On the 108th day, some of the microspheres were broken into thin sheets and these microspheres were hollow, whereas the other part of the microsphere is completely broken in two with hollow multi-cavity structure. However, the PLLA85/15 microspheres had no obvious change on Day 45 while only a small number of them had rough surface and many small pits on Day 108. The reason was that the PLLGA75/25 was an amorphous polymer with a “softer” chain than PLLGA85/15. The thinner polymer layer was not sufficiently supported during continuous degradation and thus collapsed and ruptured as a whole, whereas the thicker polymer matrix with a few microspheres appears as a whole fracture. However, the molecular weight of PLLGA85/15 microspheres was higher, the material had a certain degree of crystallinity [33], and its chain rigidity was stronger than that of PLLGA75/25. To sum up, according to the changes in the morphology of the microspheres at different time points, the order of the degradation rate could be determined as follows: PLLA-0.62 > PLLA-PEG-PLLA > PLLGA75/25 > PLLA-1.18 > PLLGA85/15 ≈ PLLA-1.39, PLLA-3.80 and PLLA-6.89, which was consistent with the results of molecular weight changes.

### MTT assay

In order to evaluate the biocompatibility of the dermal fillers, the cell viability of L929 cells after incubation with eight dermal fillers at different concentrations (measured by the concentration of polymer) for 24, 48 and 72 h was studied by an MTT assay. As shown in Supplementary Fig. S2, the cell viabilities were higher than 80%, suggesting that these poly (aliphatic ester)-based materials were non-toxic.

### *In vivo* collagen regeneration

Encouraged by the *in vitro* degradation results of these eight polymer microspheres, five dermal fillers (PLLA-PEG-PLLA, PLLA-1.39, PLLGA85/15, PLLA-3.80 and PLLA-6.89) were selected for animal experiments considering that polymeric microspheres with slower degradation rates are better for further application in aesthetic medicine. Dermal fillers were subcutaneously injected into the back skin of healthy rabbits and the health condition and skin status were monitored. Saline was injected and used as a control. In the whole observation period (13 months), rabbits in the saline and experimental groups showed normal growth and larger body size with time. The skin on their back was normal, without infection, redness, erythema, mass or edema. For example, the H&E staining images of back slices in saline group were displayed in Supplementary Fig. S3. The dermis, subcutaneous and muscular tissues showed no lesion.

In order to evaluate the biodegradability and inflammatory response of dermal fillers, the skin tissues of corresponding rabbits at different time points (0.5, 1.5, 2.5, 4, 6, 9 and 13 months) were fixed and stained with H&E for histological examination. Meanwhile, to explore the capability to promote collagen regeneration, Masson trichrome, CD68, Col. I and Col. III immune fluorescence staining were performed to visualize collagen, macrophages, Type I and Type III collagens, respectively.

**Figure 5.**
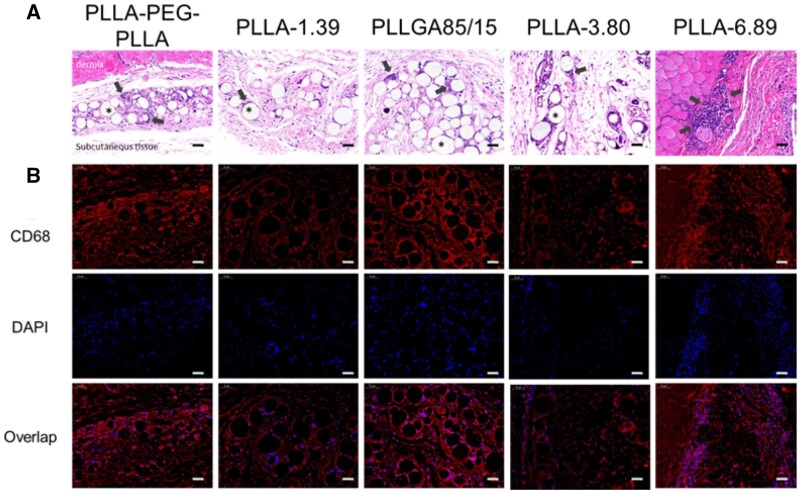
H&E (A) and Immunofluorescence staining (B) images of dermal or subcutaneous tissue at 0.5 months after injection. The scale bar is 50 μm. * represents the microspheres (the blank holes) and the black arrows point at infiltrating immune cells.

Figure 5 displays the stained images of back tissue at 0.5-month post-injection. In the H&E staining images, polymeric microspheres were distributed in loose connective tissue and surrounded by some macrophages and lymphocytes. PLLA-1.39, PLLGA85/15 and PLLA-3.80 remained spherical morphology. However, some of PLLA-PEG-PLLA microspheres were elliptic or crescent, surrounded by the most infiltrated cells, implying relatively rapid degradation; Furthermore, multinucleated giant cells appeared. PLLA-6.89 microspheres were hardly observed in the subcutaneous tissues, but only a large number of cell infiltration in the dermis were captured, which was probably due to the severe inflammatory response caused by the omission of few dermal fillers in the dermis when the needle was withdrawn at the end of injection. Immunofluorescence staining of CD68 further confirmed that macrophages surrounded the microspheres, and the CD68 fluorescence signals in PLLA-PEG-PLLA and PLLGA85/15 groups were the strongest due to a larger change in pH of surroundings caused by quickly releasing acidic degradation products, thus leading to the strongest inflammation response [34]. A large number of nuclei were also found in PLLA-6.89 group in 4',6-diamidino-2-phenylindole (DAPI) staining photographs.

**Figure 6.**
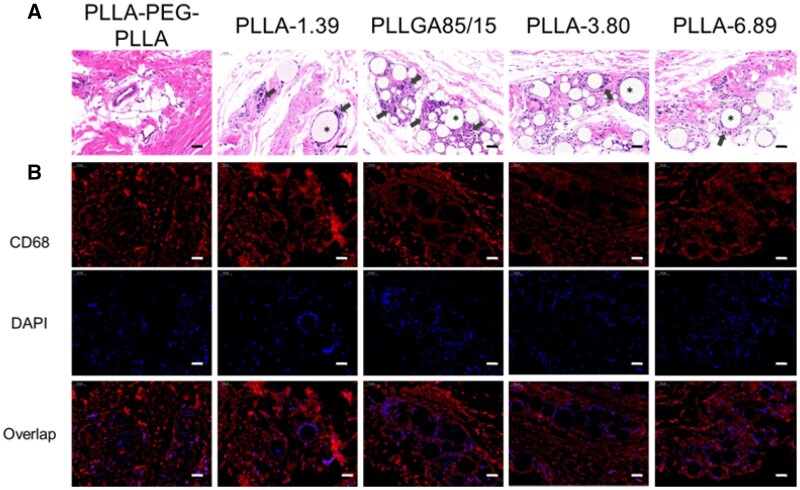
H&E (**A**) and Immunofluorescence staining (**B**) images of dermal or subcutaneous tissue at 1.5 months after injection. The scale bar is 50 μm. * represents the microspheres (the blank holes) and the black arrows point at infiltrating immune cells.

The staining images of the tissue sections at 1.5 months after injection are shown in Fig. 6. The H&E staining images demonstrated that only PLLA-PEG-PLLA microspheres were absorbed. The microspheres infiltrated by immune cells were observed in other groups. The blue nuclei stained with hematoxylin around the PLLGA85/15 microspheres were the most, indicating that the cell density was the highest. Some PLLGA85/15 microspheres had become irregular stripes, indicating deeper degradation. Similarly, the fluorescence signals of CD68 and DAPI were around the microspheres, and the fluorescence intensities in the PLLGA85/15 group was strong.

**Figure 7.**
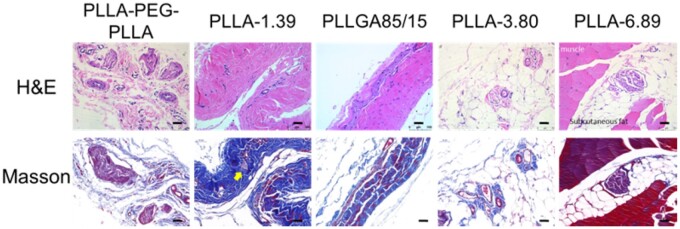
H&E and Masson trichrome staining images of subcutaneous tissue at 2.5 months after injection. The scale bar is 50 μm.

The staining images of back tissues at 2.5 months after injection are shown in Fig. 7. When compared with the results at previous time points, the fibrous capsule in PLLA-PEG-PLLA group became larger and the number of fibrous capsules increased significantly. A small number of fibroblasts were found in collagen fibers and fat lobules in PLLGA85/15, PLLA-3.80 and PLLA-6.89 groups, which may be caused by the degradation of microspheres with small particle sizes in each sample and the inclusion of fibroblasts, and this observation at around 3 months after injection was coincided with that reported in the literature [10]. Individual microspheres (yellow arrow in Masson trichrome staining images) could be seen ‘embedded’ in collagen fibers.

**Figure 8.**
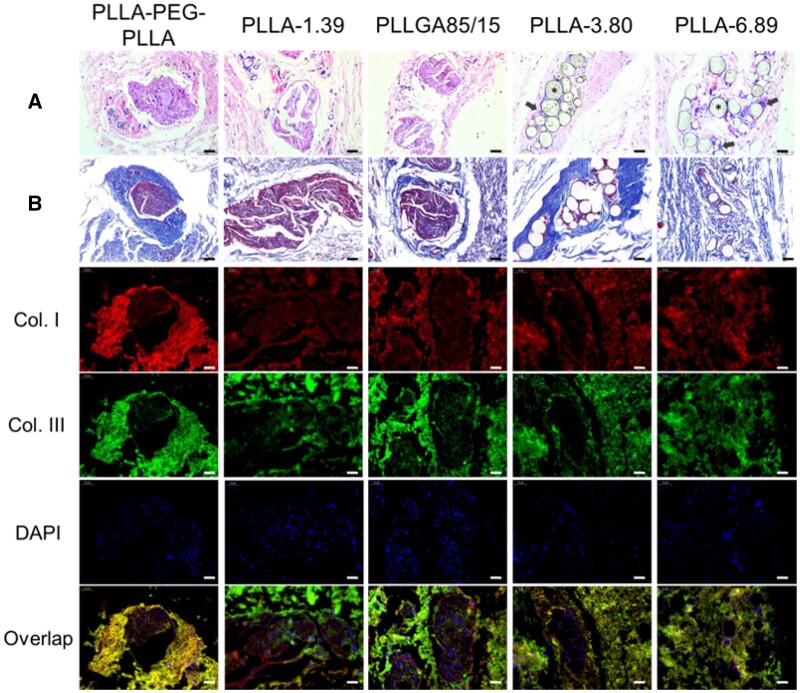
H&E (**A**), Masson trichrome (**B**) and immunofluorescence staining images of subcutaneous tissue at 4 months after injection. The scale bar is 50 μm. * represents the microspheres (the blank holes) and the black arrows point at infiltrating immune cells.

Figure 8 displays the staining images of subcutaneous tissue at 4 months after injection. H&E staining results showed that only PLLA-3.80 and PLLA-6.89 microspheres were observed in the subcutaneous tissues, and pores were observed on the surface of some microspheres. CD68 immunofluorescence staining images (Supplementary Fig. S4) showed that macrophages and giant cells still infiltrated around these two kinds of microspheres. In the groups of PLLA-PEG-PLLA, PLLA-1.39 and PLLGA85/15, the existence of multiple fibrous capsules were observed. Moreover, in the masson trichrome staining images, the fibrous capsule in the PLLA-PEG-PLLA sample was surrounded by thicker collagen fibers, and the color of the blue parts around PLLA-3.80 and PLLA-6.89 microspheres became deeper, indicating the new collagen fibers. In the immunofluorescence double-staining images, Type I collagen was mainly contained in the fibrous capsule of PLLA-PEG-PLLA, PLLA-1.39 and PLLGA85/15 samples, whereas Type III collagen was mostly contained in the outer part. PLLA-3.80 and PLLA-6.89 microspheres were surrounded by Type I collagen.

**Figure 9.**
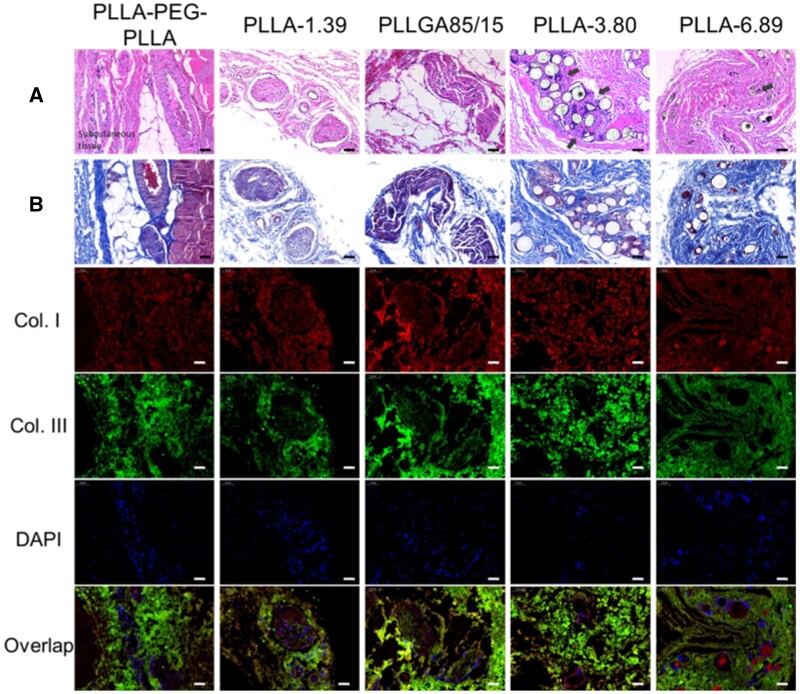
H&E (**A**), Masson trichrome (**B**) and immunofluorescence staining images of subcutaneous tissue at 6 months after injection. The scale bar is 50 μm. * represents the microspheres (the blank holes) and the black arrows point at infiltrating immune cells.

In Fig. 9, the staining images of subcutaneous tissue at 6 months after injection were shown. Multiple fibrous capsules and vascular structures were observed in the PLLA-PEG-PLLA, PLLA-1.39 and PLLGA85/15 groups. New collagen was around these fibrous capsules in the Masson trichrome staining images. H&E and Masson trichrome staining results showed that the inflammatory response of PLLA-3.80 microsphere was stronger, where more macrophages and giant cells and new collagen were around the microsphere. Immunofluorescence staining of CD68 (Supplementary Fig. S5) showed enhanced red signal around the PLLA-3.80 microsphere, and the DAPI-labeled nuclei clustered to form several bright blue clusters. Moreover, in H&E and Masson staining images, the degradation of some PLLA-3.80 microspheres was aggravated, showing semicircle or large holes on the surface. PLLA-6.89 microsphere remained spherical and was surrounded by macrophages and new collagen, but the inflammatory response induced by PLLA-6.89 microsphere was weaker than that by PLLA-3.80. In the immunofluorescence double-staining images of collagen, Type I collagen was mainly located inside the fibrous capsules of PLLA-PEG-PLLA, PLLA-1.39 and PLLGA85/15 groups while most Type III collagen outside the capsules. Similar to the results at the 4 months after injection, PLLA-3.80 and PLLA-6.89 microspheres were surrounded by Type I, and Type III collagens near them increased.

**Figure 10.**
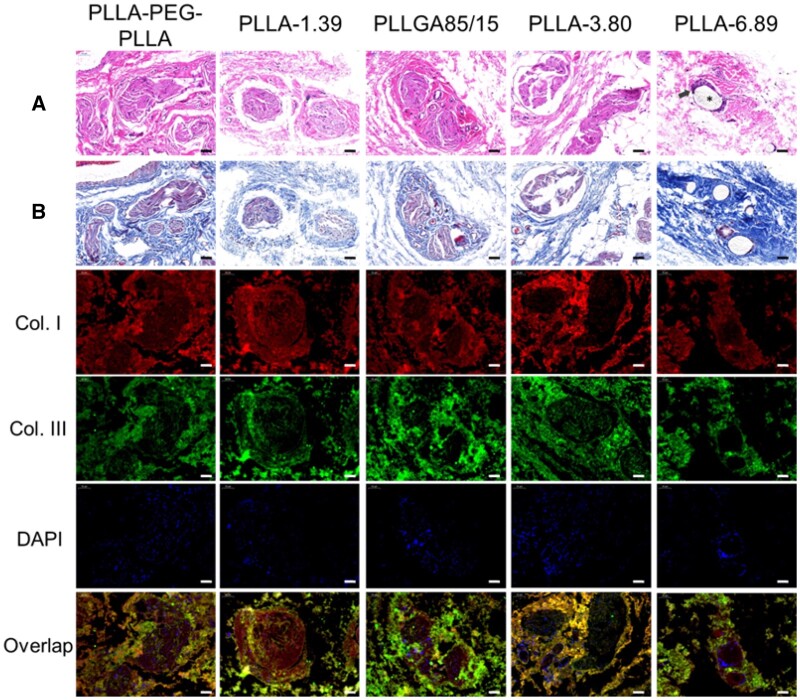
H&E (**A**), Masson trichrome (**B**) and immunofluorescence staining images of subcutaneous tissue at 9 months after injection. The scale bar is 50 μm. * represents the microspheres (the blank holes) and the black arrows point at infiltrating immune cells.

The staining results at 9 months after subcutaneous injection are shown in Fig. 10. Only PLLA-6.89 microspheres were observed in the subcutaneous tissues and CD68 immunofluorescence staining (Supplementary Fig. S6) showed that the microspheres were surrounded by a small number of macrophages and giant cells. The result that PLLA-3.80 microspheres were ‘disappeared’ at 9 months after injection was consistent with that in the literature [11]. Immunofluorescence staining images showed that they were surrounded by Type I collagen. H&E staining images of the other four groups exhibited multiple fibrous capsules and peripheral new collagen as well as vascular structures nearby. When compared with the previous time points, Type I collagen around the fibrous capsules in the groups of PLLA-PEG-PLLA and PLLA-1.39 increased. In the groups of PLLGA85/15 and PLLA-3.80, Type I collagen increased at the inner part of the fibrous capsules, whereas Type III collagen dominated the outer part.

**Figure 11.**
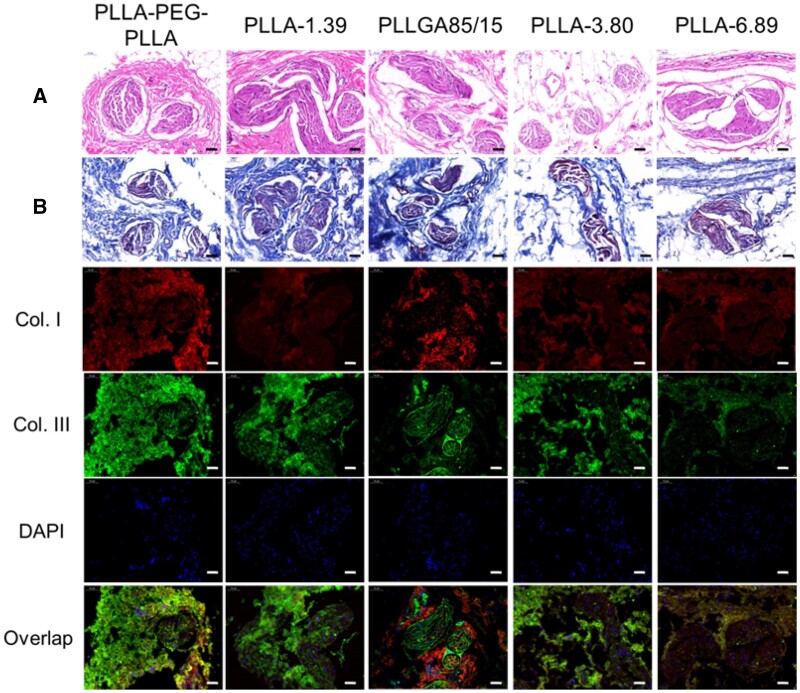
H&E (**A**), Masson trichrome (**B**) and immunofluorescence double staining images of subcutaneous tissue at 13 months after injection. The scale bar is 50 μm.

The staining results at 13 months after injection are shown in Fig.11. No microspheres were observed in all groups, indicating that all polymer microspheres could be degraded in 13 months. Immunofluorescence staining images showed that the Type III collagen contents inside and outside the fibrous capsule in all the samples except PLLA-6.89 group increased compared with those in previous time points. However, the fluorescence intensity of Type I and Type III collagens in PLLA-6.89 group was lower than that in other groups. The weak ability to induce collagen regeneration of PLLA-6.89 group was due to the slower degradation rate.

### Foreign body reaction classification and semi-quantitative analysis of dermal fillers

**Figure 12.**
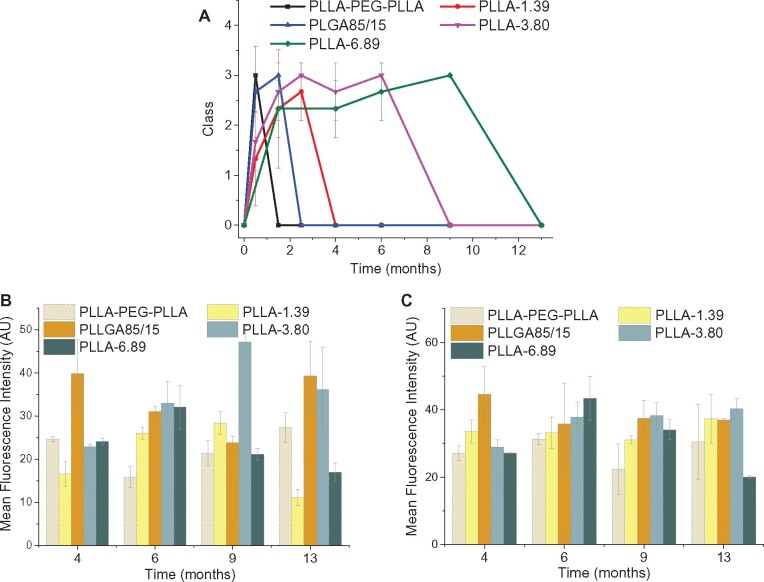
Average grades of foreign body reaction (**A**) and semi-quantitative analysis for Type I collagen (**B**), Type III collagen (**C**) regeneration induced by the five dermal fillers at each time point.

According to H&E staining images of each sample at each time point, the foreign body reaction classification criteria established by Duranti *et al.* was used to assess the foreign body reaction; the results are shown in Fig. 12A. PLLA-PEG-PLLA and PLLGA85/15 dermal fillers showed the fastest speed to induce foreign body reaction, reaching Grade III at 0.5 and 1.5 months after injection, respectively. The inflammatory response induced by PLLA-3.80 dermal filler was relatively weak. The average grade of PLLA-3.80 was higher than PLLA-1.39 at 0–2.5th month. However, due to the slower degradation rate, the dermal filler from PLLA-3.80 group was ‘disappeared’ at 9 months after injection, whereas PLLA-1.39 dermal filler was ‘disappeared’ as early as 4 months after injection. The grade of PLLA-6.89 dermal filler was the latest to reach III at 9 months after injection.

The semi-quantitative analysis of collagen immunofluorescence staining images were further performed by ImageJ and the results were shown in Fig. 12B and C. PLLA-3.80 and PLLGA85/15 elicited more generation of Type I and Type III collagens. The most generation of Type I collagen by PLLA-3.80 was observed at 9th month while PLLGA85/15 could maintain a high-level Collagen I induction through the observation periods (4–13 months). PLLA-PEG-PLLA and PLLA-1.39 groups showed much less generation of Type I collagen. Although having a higher polymer molecular weight, PLLA-6.89 group displayed less collagen regeneration and the level was remarkably decreased from 6 to 13 months post injection. Taking together, these results manifested that the dermal fillers with fast degradation properties like PLLA-PEG-PLLA and PLLA85/15 could maintain the regenerative collagen for a relatively long time but induce acute inflammation response; although the inflammation response induced by PLLA-6.89 with slow degradation was weak and late, it did not achieve the expected neocollagenesis effect. Therefore, only the dermal fillers with moderate degradation such as PLLA-3.80 could balance the collagen generation and inflammation response.

## Conclusions

Eight poly (aliphatic ester)-based microspheres with different molecular weights, chemical structures and architectures were prepared. Microspheres with size range of 20–100 μm and spherical morphology were obtained by emulsion-solvent evaporation method. Both 25 and 50 kGy γ-ray were employed to sterilize microspheres, which reduced the molecular weights of polymers but did not change the size and morphology of microspheres. These poly (aliphatic ester) microspheres showed excellent cell compatibility and biodegradability. The degradation study of dermal fillers in rabbits established the *in vivo* biodegradability and collagen regeneration induced by thermal fillers. New collagen, especially Type I collagen mainly existed around the microsphere and inside the fibrous capsules, whereas Type III collagen dominated outside the fibrous capsules. The degradation rate of microspheres had a strong correlation with the foreign body reaction induced by microspheres: the faster the degradation rate of microspheres, the earlier they could cause obvious inflammatory response and more quickly fibrous capsules were formed, so the weaker the effect of maintaining soft tissue volume would be. The foreign body reaction and stimulating collagen regeneration caused by PLLA-6.89 microspheres with the slowest degradation rate were also relatively weak. But the PLLA-3.80 microsphere with a moderate degradation rate showed late foreign body reaction and obvious collagen regeneration, and its slow degradation rate also enabled an increase of soft tissue volume, which maintained a long-term aesthetic medicine effect.

## Supplementary data

Supplementary data are available at *REGBIO* online.

## Funding

This work was supported by the National Science Foundation of China (51773130), Sichuan Science and Technology Program (2019JDRC0098), SCU-Enterprise Joint Project (18H0350), and Sichuan Province Health Department (18P J553).

*Conflict of interest statement*. None declared.

## Supplementary Material

rbab042_Supplementary_Data

## References

[1] Akinbiyi T, Othman S, Familusi O et al Better results in facial rejuvenation with fillers. Plast Reconstr Surg Glob Open 2020;8:e2763. 33173655 10.1097/GOX.0000000000002763 PMC7647625

[2] Pan Y, Hao Y, Xiao Y et al Injectable soft tissue nano/micro fillers for facial reconstruction. J Biomed Nanotechnol 2021;17:1. 33653493 10.1166/jbn.2021.3011

[3] Joseph JH, Eaton LL, Cohen SR. Current concepts in the use of bellafill. Plast Reconstr Surg 2015;136:171S–9S. 26441096 10.1097/PRS.0000000000001839

[4] Lee JC, Lorenc ZP. Synthetic fillers for facial rejuvenation. Clin Plast Surg 2016;43:497–503. 27363763 10.1016/j.cps.2016.03.002

[5] Danny V. Soft-tissue augmentation and the role of poly-L-lactic acid. Plast Reconstr Surg 2006;118:S46–54. 10.1097/01.prs.0000234846.00139.74 16936544

[6] Stein P, Vitavska O, Kind P et al The biological basis for poly-L-lactic acid-induced augmentation. J Dermatol Sci 2015;78:26–33. 25703057 10.1016/j.jdermsci.2015.01.012

[7] Rotunda AM, Narins RS. Poly-L-lactic acid: a new dimension in soft tissue augmentation. Dermatol Ther 2006;19:151–8. 16784514 10.1111/j.1529-8019.2006.00069.x

[8] Butterwick K, Lowe NJ. Injectable poly-L-lactic acid for cosmetic enhancement: learning from the European experience. J Am Acad Dermatol 2009;61:281–93. 19615539 10.1016/j.jaad.2008.11.881

[9] Liu J, Tagami T, Ozeki T. Fabrication of 3D-printed fish-gelatin-based polymer hydrogel patches for local delivery of PEGylated liposomal doxorubicin. Mar Drugs 2020;18:325. 10.3390/md18060325 PMC7344981 32575787

[10] André P, Villain F. Free radical scavenging properties of mannitol and its role as a constituent of hyaluronic acid fillers: a literature review. Int J Cosmet Sci 2017;39:355–60. 28027572 10.1111/ics.12386

[11] Fitzgerald R, Bass LM, J Goldberg D et al Physiochemical characteristics of poly-L-lactic acid (PLLA). Aesthet Surg J 2018;38:13–7. 10.1093/asj/sjy012 29897517

[12] Lemperle G, Morhenn V, Charrier U. Human histology and persistence of various injectable filler substances for soft tissue augmentation. Aesthetic Plast Surg 2003;27:354–66. 14648064 10.1007/s00266-003-3022-1

[13] Goldberg D, Guana A, Volk A et al Single-arm study for the characterization of human tissue response to injectable poly-L-lactic acid. Dermatol Surg 2013;39:915–22. 23464798 10.1111/dsu.12164

[14] Li J, Zhang X, Zhao M et al Tumor-pH-sensitive PLLA-based microsphere with acid cleavable acetal bonds on the backbone for efficient localized chemotherapy. Biomacromolecules 2018;19:3140–8. 29883542 10.1021/acs.biomac.8b00734

[15] Cheng F, Peng X, Meng G et al Poly(ester-thioether) microspheres co-loaded with erlotinib and α-tocopheryl succinate for combinational therapy of non-small cell lung cancer. J Mater Chem B 2020;8:1728–38. 32022097 10.1039/c9tb02840d

[16] Cheng F, Su T, Pu Y et al Polymer structure-guided self-assisted preparation of Poly(ester-thioether)-based hollow porous microspheres and hierarchically interconnected microcages for drug release. Macromol Biosci 2019;19:1900171. 10.1002/mabi.201900171 31486275

[17] Clarke N, Yusuf K, Laurencin C. Nanofiber–microsphere (nano-micro) matrices for bone regenerative engineering: a convergence approach toward matrix design. Regen Biomater 2014;1:3–9. 26816620 10.1093/rb/rbu002 PMC4669008

[18] Chen Y, Huang J, Liu J et al Tuning filament composition and microstructure of 3D-printed bioceramic scaffolds facilitate bone defect regeneration and repair. Regen Biomater 2021;8:rbab007. 33738121 10.1093/rb/rbab007 PMC7955715

[19] Kwon T, Han SW, Yeo IK et al Biostimulatory effects of polydioxanone, poly-d, l lactic acid, and polycaprolactone fillers in mouse model. J Cosmet Dermatol 2019;18:1002–8. 30985064 10.1111/jocd.12950

[20] Duranti , Salti G, Bovani R et al Effects of monopolar radiofrequency treatment over soft-tissue fillers in an animal model. Dermatol Surg 1998;24:1317–25.9865196

[21] Crowley J, Kream E, Fabi S et al Facial rejuvenation with fat grafting and fillers. Aesthet Surg J 2021;41:S31–8. 34002771 10.1093/asj/sjab014

[22] Pradeesh TS, Sunny MC, Varma HK et al Preparation of microstructured hydroxyapatite microspheres using oil in water emulsions. Bull Mater Sci 2005;28:383–90.

[23] Katou H, Wandrey AJ, Gander B. Kinetics of solvent extraction/evaporation process for PLGA microparticle fabrication. Int J Pharm 2008;364:45–53. 18782610 10.1016/j.ijpharm.2008.08.015

[24] Ramazani F, Chen W, van Nostrum CF et al Strategies for encapsulation of small hydrophilic and amphiphilic drugs in PLGA microspheres: state-of-the-art and challenges. Int J Pharm 2016;499:358–67. 26795193 10.1016/j.ijpharm.2016.01.020

[25] Na N, Guo H, Zhang S et al In vitro and in vivo acute toxicity of fenpyroximate to flounder *Paralichthys olivaceus* and its gill cell line FG. Aquat Toxicol 2009;92:76–85. 19185358 10.1016/j.aquatox.2008.12.006

[26] Xing R, Wang X, Zhang C et al Characterization and cellular uptake of platinum anticancer drugs encapsulated in apoferritin. J Inorg Biochem 2009;103:1039–44. 19501911 10.1016/j.jinorgbio.2009.05.001

[27] Xia Y, Pack DW. Uniform biodegradable microparticle systems for controlled release. Chem Eng Sci 2015;125:129–43.

[28] Li SM, Garreau H, Vert M. Structure-property relationships in the case of the degradation of massive aliphatic poly-(α-hydroxy acids) in aqueous media, Part 1 Poly (DL-lactic acid). J Mater Sci: Mater Med 1990;1:123–30.

[29] Therin M, Christel P, Li S et al In vivo degradation of massive poly(α-hydroxy acids): validation of In vitro findings. Biomaterials 1992;13:594–600. 1391406 10.1016/0142-9612(92)90027-l

[30] Anderson JM, Shive MS. Biodegradation and biocompatibility of PLA and PLGA microsphere. Adv Drug Deliv Rev 2012;64:72–82. 10.1016/s0169-409x(97)00048-3 10837562

[31] Liu Y, Wang Y, Zhang M et al A new insight into formation of 3D porous biomaterials. J Mater Sci 2021;56:3404–10.

[32] Tsuji H, Nakahara K. Poly(L-lactide). IX. Hydrolysis in acid media. J Appl Polym Sci 2002;86:186–94.

[33] Pimenta de Melo L, Salmoria G, Fancello E et al Effect of injection molding melt temperatures on PLGA craniofacial plate properties during in vitro degradation. Int J Biomater 2017;2017:1256537–11. 29056968 10.1155/2017/1256537 PMC5606095

[34] Weir NA, Buchanan FJ, Orr JF et al Degradation of poly-L-lactide. Part 1: in vitro and in vivo physiological temperature degradation. Proc Inst Mech Eng H 2004;218:307–19. 15532996 10.1243/0954411041932782

