# Supplementary material for: In vivo inducing collagen regeneration of biodegradable polymer microspheres
Source: Regen Biomater. 2021 Jul 15;8(5):rbab042. doi: 10.1093/rb/rbab042 (PMC8364987; doi:10.1093/rb/rbab042)
Supplement: rbab042_Supplementary_Data [file rbab042_supplementary_data.docx]

Supporting Information of

***In vivo* inducing collagen regeneration of biodegradable polymer microspheres**

Yixin Zhang,^a^ Hanwen Liang,^b^ Qian Luo,^b^ Jianlin Chen,*^b^ Nan Zhao,^c^ Wenxia Gao,^d^ Yuji Pu,^e^ Bin He,*^e^ Jing Xie*^f^

^a^ School of Smart Health, Chongqing College of Electronic Engineering, Chongqing, 401331, China

^b^ School of Laboratory Medicine, Sichuan Provincial Engineering Laboratory for Prevention and Control Technology of Veterinary Drug Residue in Animal-origin Food, Chengdu Medical College, Chengdu, 610500, China

^c^ Puliyan (Nanjing) Medical Science & Technology Co. LTD, Nanjing, 211500, China

^d^ College of Chemistry and Materials Engineering, Wenzhou University, Wenzhou 325027, China

^e^ National Engineering Research Center for Biomaterials, Sichuan University, Chengdu 610064, China

^f^ Department of Stomatology, the First Affiliated Hospital of Wenzhou Medical University, Wenzhou 325000, China.

Table S1. Feeding weights of initiators and monomers in the synthesis of different polymers.

| Polymers | Feeding weight of initiators (g) | | Feeding weight of monomers (g) | | |
| --- | --- | --- | --- | --- | --- |
|  | DL-lactic acid | PEG | | L-LA | GA |
| PLLA-0.62 | 0.18 | -- | | 20 | -- |
| PLLA-1.18 | 0.06 | -- | | 20 | -- |
| PLLA-1.39 | 0.036 | -- | | 20 | -- |
| PLLA-6.89 | -- | -- | | 20 | -- |
| PLLGA75/25 | 0.055 | -- | | 14.4 | 3.9 |
| PLLGA85/15 | 0.05 | -- | | 14.4 | 2.05 |
| PLLA-PEG-PLLA | -- | 0.62 | | 9.73 | -- |


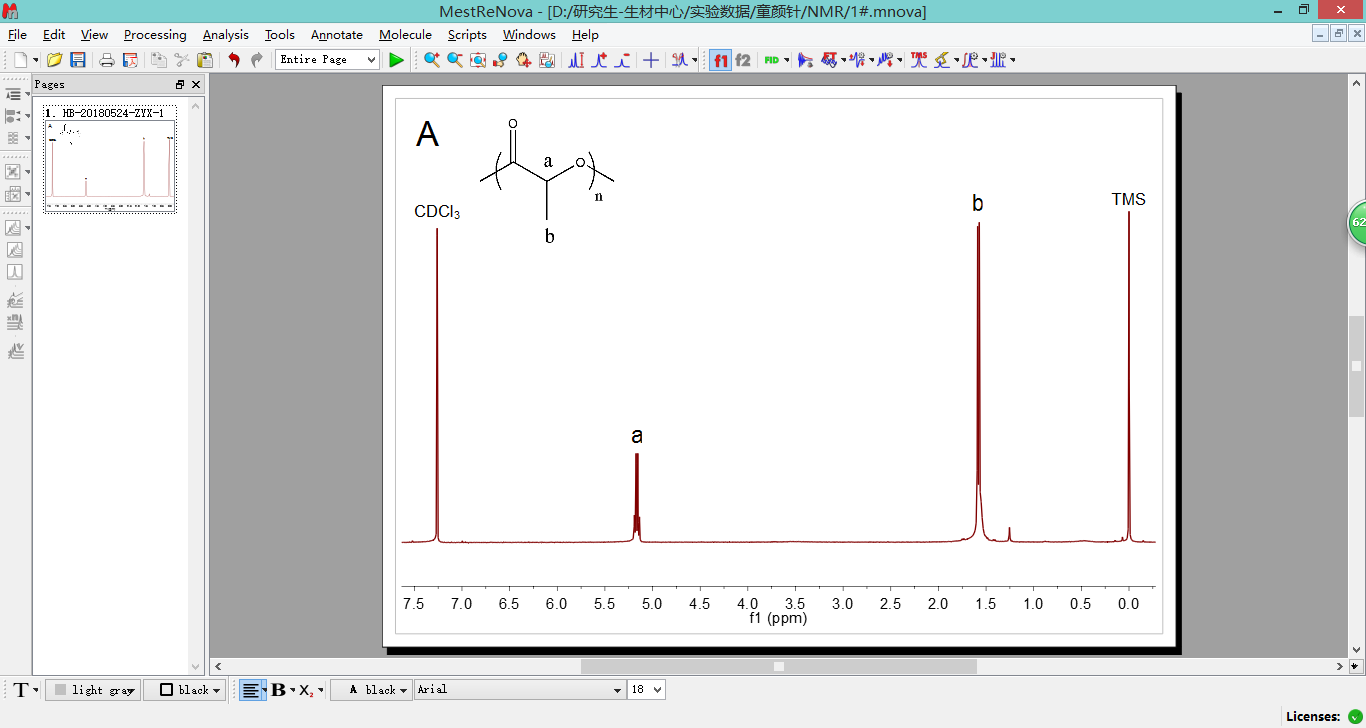

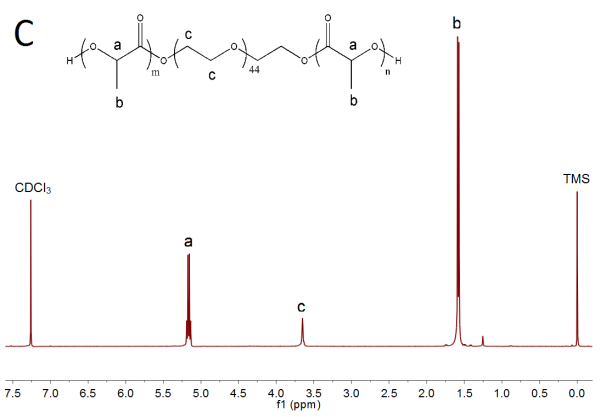


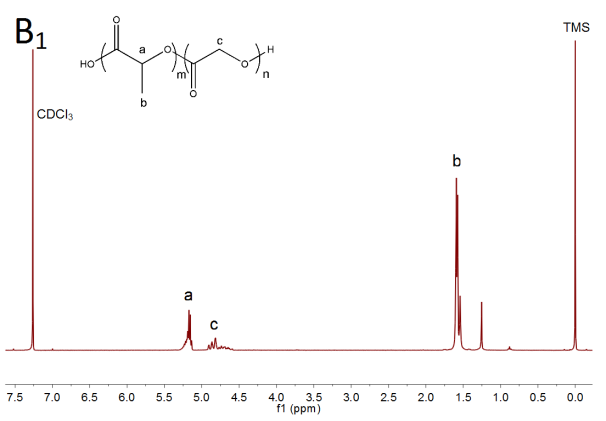

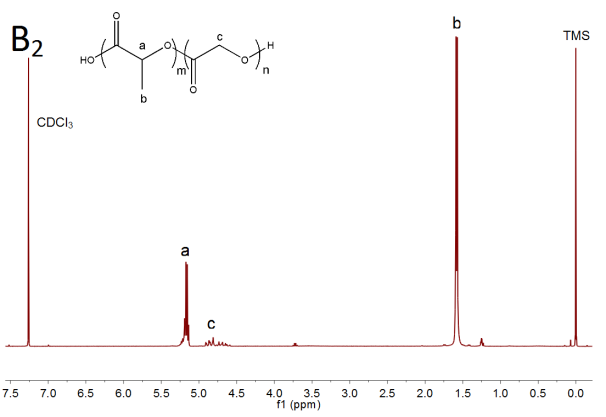


Fig.S1. ^1^H NMR spectra of PLLA-0.62 (A), PLLGA75/25 (B_1_), PLLGA85/15 (B_2_), and PLLA-PEG-PLLA (C).

Fig.S1A showed the ^1^H NMR spectrum of PLLA-0.62. The signals at δ=1.6 (b) and 5.2 (a) ppm correspond to the protons of methyl and tertiary carbon, respectively, indicating that the chemical structure conforms to the expected design. ^1^HNMR spectra of PLLGA75/25 and PLLGA85/15 were depicted in Fig.S1 (B_1_) and Fig.S1 (B_2_), respectively. The signals at δ=1.6(b) and 5.2 (a) ppm in both figures assigned to the protons of PLLA segments while the signal at δ=4.8 (c) ppm corresponded to the protons of PGA segments. And in Fig.S1 (B_1_), the molar ratio of two monomers (LA/GA) was 77/23 according to the peak area integral ratio of signal peaks at 1.6 (b) and 4.8 (c) ppm. Similarly, the molar ratio between LA and GA in Fig.S1 (B_2_) was 84/16. These values indicated that the molar ratios of two PLLGA materials conformed the anticipated design. Fig.S1 (C) displayed the ^1^H NMR spectra of tri-block copolymer PLLA-PEG-PLLA. The signals at δ=1.6(b) and 5.2 (a) ppm corresponded to the protons of PLLA segments while the signal at δ=3.65 (c) ppm assigned the protons of PEG segment. According to the peak area ratio of signal at 3.65 (c) and 5.2 (a) ppm, the M_n_ of the tri-block copolymer was calculated to be 32.37 kDa.

Table S2 The preparation conditions of different polymeric microspheres and their SEM sizes.

| Polymers | C* (mg/mL) | PVA (%) | Mechanical stirring speed (rpm) | High speed homogenizer speed (rpm) | Temperature (ºC) | Size (µm) |
| --- | --- | --- | --- | --- | --- | --- |
| PLLA-0.62 | 80 | 0.5 | 1100 | -- | 30 | 20-80 |
| PLLA-1.18 | 55 | 1 | 800 | -- | 25 | 30-90 |
| PLLA-1.39 | 50 | 0.3 | 1500 | -- | 25 | 20-85 |
| PLLA-3.80 | 50 | 0.2 | -- | 4300 | 15 | 20-90 |
| PLLA-6.89 | 33 | 0.2 | -- | 5000 | 15 | 5-100 |
| PLLGA75/25 | 30 | 0.2 | 1500 | -- | 25 | 20-75 |
| PLLGA85/15 | 55 | 1 | 800 | -- | 20 | 20-75 |
| PLLA-PEG-PLLA | 100 | 0.2 | 1400 | -- | 10 | 50-100 |

*: The polymeric concentration in oil phase.

Table S3 Number-average molecular weights (*M*_n_) and PDI of PLLA-3.80 microspheres before and after ray irradiation.

|  | 0 kGy | 25 kGy | 50 kGy |
| --- | --- | --- | --- |
| *M*_n_ (kDa) | 391 | 157 | 42.9 |
| PDI | 1.71 | 1.39 | 1.48 |

Fig. S2. Cytotoxicity of different polymeric microsphere dermal fillers.

In Fig. S2, the results showed that under most conditions (material concentration and incubation time), the cell viability was above 90%. With the extension of incubation time, it generally decreased. However, the cell viability of all dermal fillers was above 80% even when the polymer concentration was up to 500 μg/mL and incubation time was 72 h.


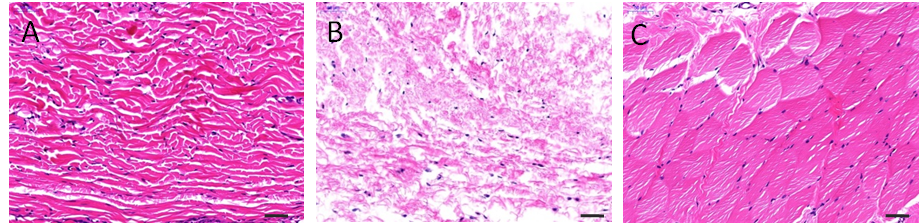


Fig. S3. H&E staining images of dermal (A), subcutaneous (B) and muscular (C) tissue of the rabbit from saline group (control group). The scale bar is 50 μm.


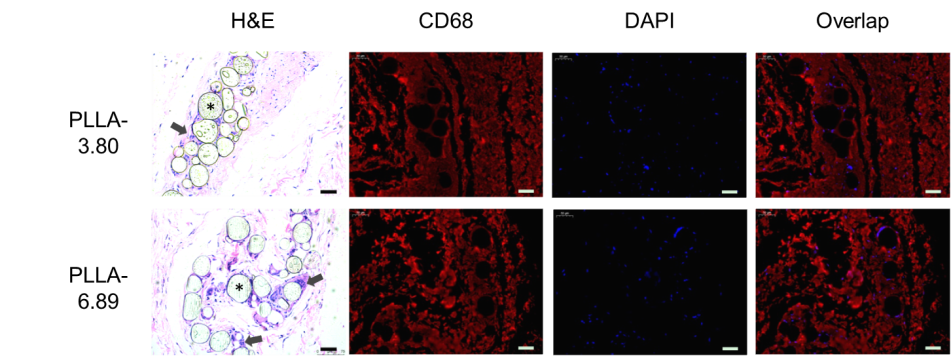


Fig. S4. H&E and CD68 immunofluorescence staining images of subcutaneous tissue from PLLA-3.80 and PLLA-6.89 samples at 4 months after injection. The scale bar is 50 μm. * represents the microspheres (the blank holes) and the black arrows point at infiltrating immune cells.


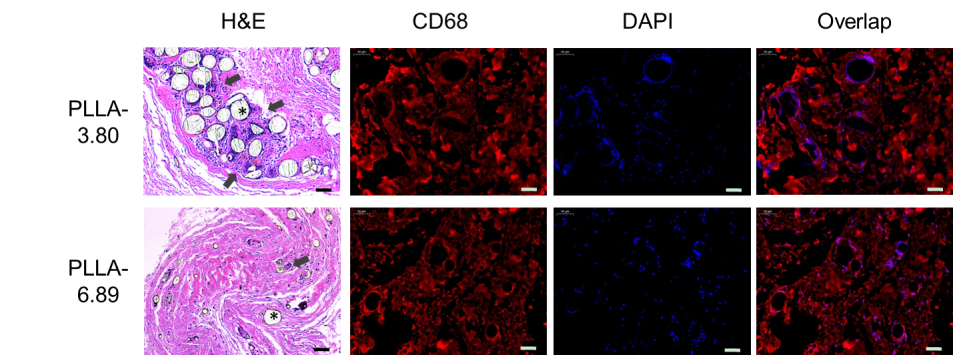


Fig. S5. H&E and CD68 immunofluorescence staining images of subcutaneous tissue from PLLA-3.80 and PLLA-6.89 samples at 6 months after injection. The scale bar is 50 μm. * represents the microspheres (the blank holes) and the black arrows point at infiltrating immune cells.


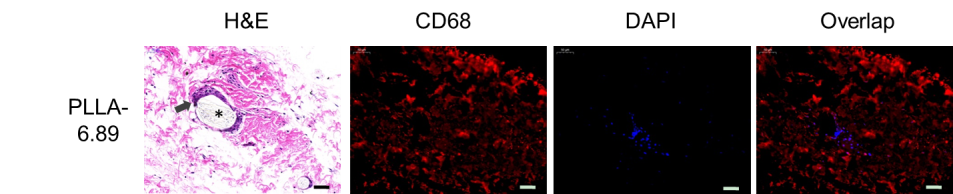


Fig. S6. H&E and CD68 immunofluorescence staining images of subcutaneous tissue from PLLA-3.80 and PLLA-6.89 samples at 9 months after injection. The scale bar is 50 μm. * represents the microspheres (the blank holes) and the black arrows point at infiltrating immune cells.
